# Supplementary material for: Human Natural Antibodies Recognizing Glycan Galβ1-3GlcNAc (LeC)
Source: Int J Mol Sci. 2020 Sep 5;21(18):6511. doi: 10.3390/ijms21186511 (PMC7554730; doi:10.3390/ijms21186511)
Supplement: Supplementary file 1 [file ijms-21-06511-s001.zip › ijms-905383-Supplementary Figures.pdf]

International Journal of
Molecular Sciences

MDPI

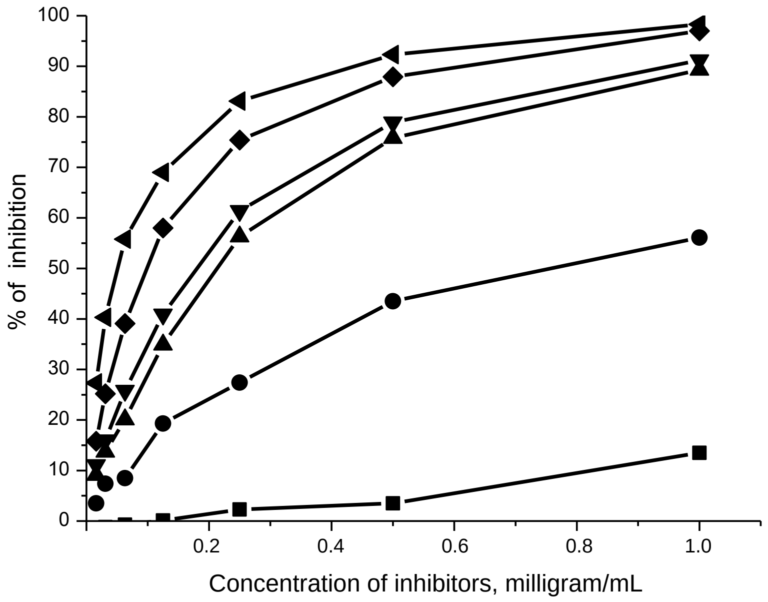

Figure S1. Inhibition of human anti-Le^C Abs (isolated from blood serum) binding to Le^C-PAA (as a coating antigen in ELISA) with GlcNAcβ1-OSer and other glycosides, top-down: Galβ1-3GlcNAcβ-$OCH_2CH_2CH_2NH_2$; Galβ1-3GlcNAcβ-$OCH_2CH_2NH_2$; GlcNAcβ-$OCH_2CH_2CH_2NH_2$; GlcNAcβ-$OCH_2CH_2NH_2$; GlcNAcβ-O-L-Ser(*N*Ac); GlcNAcβ-O-D-Ser(*N*Ac).

International Journal of
Molecular Sciences

MDPI

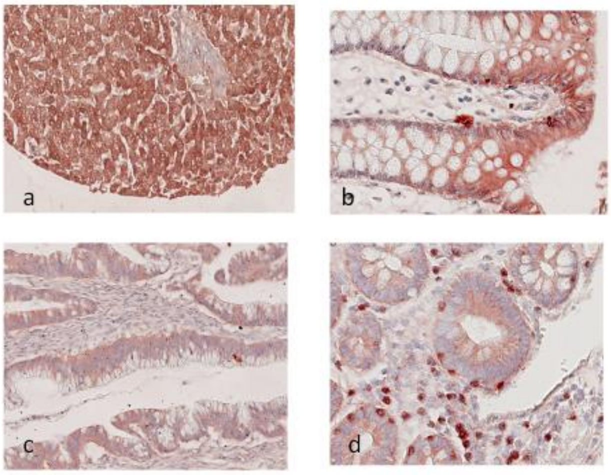

e

Tissue	Human anti-Le^{C} Abs
Pylorus, duodenum	+
Oesophagus, stomach	+
Middle colon	+
Riht colon	+
Liver	++
Pancreas	+
Endometrium	+
Ovary	+
Cervix	-
Vagina	+
Oviduct	+
Trachea	-

Figure S2. Binding of human anti-Le^{C} Abs to human tissues. Healthy tissue samples from 10 donors were used to construct a TissueMicroArray that was incubated with biotin-labeled anti-Le^{C} Abs (a-e). Binding was detected using peroxidase-labeled avidin and counterstaining performed with hematoxylin. Examples of typical staining results are shown. Liver (a), colon mucosa (b), colon carcinoma (c), normal appearing colonic mucosa adjacent to a tumor (d) and staining levels of healthy tissues (e). Blue color on the developed slides is due to hematoxyline staining of DNA/RNA containing cell structures (nuclei) and pink color is due to the bound antibodies.

International Journal of Molecular Sciences

MDPI

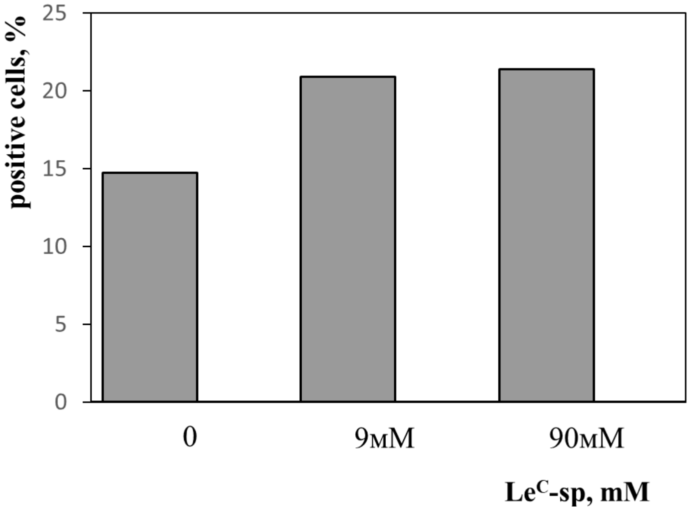

Figure S3. Interaction of human antibodies affinity-isolated using Le^{C}-Sepharose with ZR 75-1 cells (70% grown density of monolayer) in the presence of free Galβ1-3GlcNAcβ-sp (Le^{C}-sp) disaccharide, sp = $OCH_2CH_2CH_2NH_2$. Data of flow cytometry.

International Journal of *Molecular Sciences*

MDPI

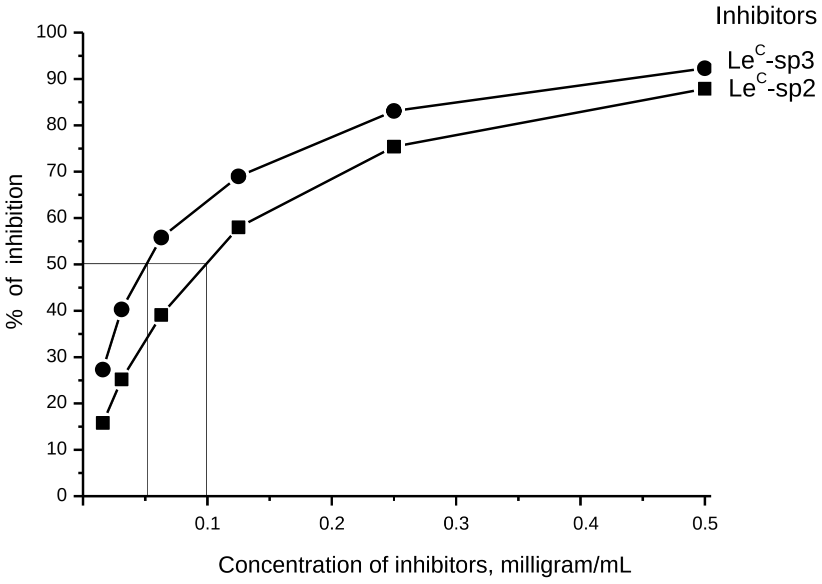

Figure S4. Evaluation of anti-Le^{C} nAbs affinity towards monomeric disaccharide Galβ1-3GlcNAc and estimate of K_d for anti-Le^{C} antibodies using inhibition ELISA. K_d was determined as a value corresponding to the molar concentration of free monomeric antigen-inhibitors Le^{C}-sp3 (Le^{C}-$OCH_2CH_2CH_2NH_2$) and Le^{C}-sp2 (Le^{C}-$OCH_2CH_2NH_2$) necessary for saturation of half of all antigen-binding sites of antibodies, i.e. for 50% inhibition of binding of antibodies to the immobilized antigen (Le^{C}-PAA): K_d = [Le^{C}] = $I_{50\%}$ (g/L) = M (g/mol), where [Le^{C}] is the molar concentration of the monomeric antigen, $I_{50\%}$ is mass concentration of free Le^{C} disaccharide necessary for 50% inhibition, M is molar mass of the disaccharide. The found values are: $I_{50\%}$ (Le^{C}-sp3) = 51 μg/mL, K_d = 1.4×10^{-4} M.

International Journal of
Molecular Sciences

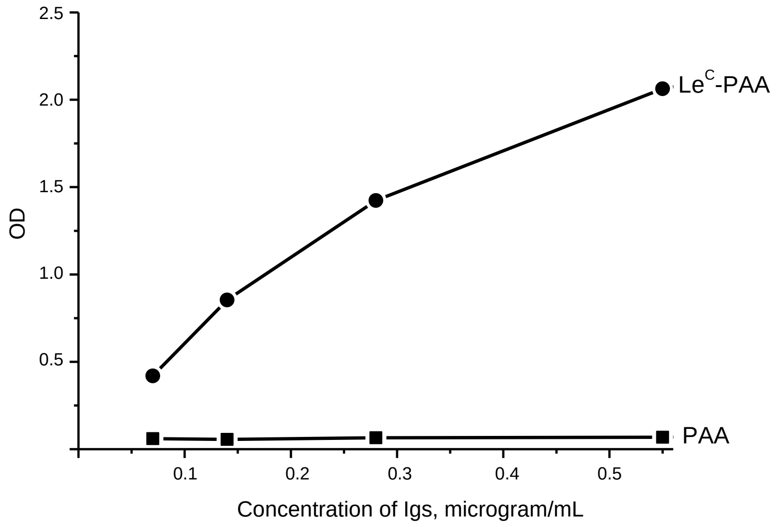

Figure S5. Affinity of the antibodies towards polymeric antigen, Le^C-PAA, estimated using ELISA. Immunological plates (NUNC MaxiSorb) were coated with Le^C-PAA. The affinity constant was evaluated as an amount of Abs equal to the concentration of Abs giving the signal intensity (OD) = 1 in the linear region of the *signal intensity vs. antibody quantity* plot, i.e. $K_d \sim$ [Abs]. The concentration of anti-Le^C antibodies (at OD = 1) was 0.2 μg/mL, i.e. K_d is equal to 8.6×10^{-10} M.
